# Trophoblast invasion biology: from normal implantation to accreta spectrum and choriocarcinoma

**DOI:** 10.1530/VB-25-0016

**Published:** 2026-04-08

**Authors:** Mishu Mangla, Rohini Motwani, Seetu Palo, Aparna Setty

**Affiliations:** ^1^ Department of Obstetrics and Gynecology, All India Institute of Medical Sciences, Hyderabad, India; ^2^ Department of Anatomy, All India Institute of Medical Sciences, Hyderabad, India; ^3^ Department of Pathology and Laboratory Medicine, All India Institute of Medical Sciences, Hyderabad, Telangana, India

**Keywords:** angiogenesis, choriocarcinoma, malignancy, matrix metalloproteinases, placenta accreta spectrum (PAS), trophoblast invasion, vascular remodeling

## Abstract

Trophoblast invasion is a finely regulated physiological process that demonstrates controlled cellular migration and vascular remodeling during human pregnancy. During normal implantation, cytotrophoblasts differentiate into extravillous trophoblasts (EVTs), which enter the maternal decidua and remodel spiral arteries, converting them into low-resistance vessels required for fetal nutrition. This balance of invasion and restraint promotes appropriate vascular adaptation at the maternal–fetal interface. Aberrations in this mechanism result in a range of pathological states, from excessive but non-malignant invasion in placenta accreta spectrum (PAS) to uncontrolled, malignant invasion in choriocarcinoma. In PAS, trophoblasts can infiltrate the myometrium due to dysregulated production of matrix metalloproteinases (MMP-2 and MMP-9), integrins, and angiogenic factors (VEGF and HIF-1α). In choriocarcinoma, trophoblastic cells activate PI3K/AKT, Wnt/β-catenin, and Notch signaling, leading to hyperproliferation, immune evasion through PD-L1, and metastatic potential. Despite differences in clinical behavior, many disorders have molecular mediators that link trophoblast invasion to vascular remodeling, angiogenesis, and immunological regulation. Emerging models, including trophoblast organoids, placental explants, and placenta-on-a-chip technologies, offer enhanced platforms to study such pathways at the cellular and molecular levels. This review highlights trophoblast invasion as a model of controlled angiogenic remodeling, providing mechanistic insight into both obstetric pathology and cancer biology. It underscores how studying placental invasion can inform therapeutic strategies targeting abnormal vascular invasion across diseases.

## Introduction

Trophoblast invasion is a hallmark of early pregnancy, ensuring implantation and placental development. During normal implantation, cytotrophoblasts differentiate into extravillous trophoblasts (EVTs) that invade the maternal decidua and remodel spiral arteries, establishing an optimal uteroplacental circulation (1). This tightly regulated process is crucial for fetal development and maternal adaptation, representing one of the most sophisticated examples of physiological invasion in human biology (2). Deviating from the normal trophoblast invasion, pathological states emerge at both ends of the spectrum. On one end lies placenta accreta spectrum (PAS), a condition in which deficient decidualization or uterine scarring permits abnormally deep invasion of trophoblasts into the myometrium (3). While trophoblastic cells in PAS remain non-malignant, their unchecked penetration leads to catastrophic obstetric complications, including life-threatening hemorrhage during delivery and often necessitates complex surgical intervention (3). On the other end of the spectrum is invasive mole and gestational choriocarcinoma, characterized by aggressive proliferation, destructive invasion as evidenced by loss of villous structure, anaplasia, and widespread vascular invasion, and a remarkable propensity for hematogenous metastasis (4). Although malignant, choriocarcinoma cells share molecular features with invasive EVTs, underscoring the blurred boundary between physiological and pathological invasion. Insights into these shared pathways provide a valuable understanding of both placental development and cancer biology (5).

Despite their contrasting clinical outcomes, PAS and choriocarcinoma share a common root in the biology of trophoblastic invasion (6). Both conditions exhibit dysregulation of extracellular matrix (ECM) degradation, angiogenesis, and immune modulation, processes that are normally tightly balanced during implantation. Hyperglycosylated human chorionic gonadotropin (H-hCG), matrix metalloproteinases (MMPs), integrins, and angiogenic factors are central mediators in all three contexts, but their regulation and outcomes differ. In PAS, invasion is excessive yet localized, reflecting loss of ‘stop’ signals from decidua and uterine stroma. In choriocarcinoma, invasion is accompanied by unchecked proliferation, genomic instability, immune evasion, and systemic dissemination, features that parallel other aggressive malignancies (7). Therefore, studying these three states together provides a unique lens into invasion biology, implantation as the physiological baseline, PAS as an intermediate pathological exaggeration, and choriocarcinoma as a malignant transformation. Recent advances in single-cell transcriptomics, spatial profiling, and *ex vivo* trophoblast models have illuminated the molecular and cellular programs underlying these conditions, but a comprehensive comparative synthesis is lacking. A comparative overview of trophoblastic invasion across normal implantation, PAS, and choriocarcinoma is presented in Table 1. This review synthesizes current evidence on trophoblastic invasion, emphasizing regulatory parallels and divergences across normal implantation, PAS, and choriocarcinoma. We highlight cellular behaviors, molecular pathways, immune microenvironmental changes, and clinical manifestations, with emphasis on how similarities and differences can inform both perinatal medicine and oncology. Understanding the continuum of trophoblast invasion may open new avenues for novel biomarker discovery, early diagnosis, and targeted therapies in both obstetric and oncologic contexts.

**Table 1:** Comparative features of trophoblastic invasion in normal implantation, PAS, and choriocarcinoma.

Feature	Normal implantation	PAS	Choriocarcinoma
Cellular and tissue level			
Depth of invasion	Controlled, limited to the inner myometrium, with remodeling of spiral arteries	Excessive, abnormally deep into the myometrium due to absent/defective decidua	Unrestricted, highly aggressive invasion into the myometrium, vessels, and distant metastasis
Trophoblast subtype	Differentiated extravillous trophoblasts (EVTs) with regulated proliferation	EVTs penetrate excessively but retain non-malignant features	Proliferative trophoblastic cells with loss of differentiation and unchecked proliferation
Molecular drivers	Balanced MMPs, integrins (α1, α5, and β1), VEGF, HIF pathways	Upregulation of MMPs, altered integrin profile, and increased angiogenic signaling	Hyperglycosylated hCG, oncogenic miRNAs, high MMP activity, VEGF, PI3K/AKT, Wnt/β-catenin activation
Immune microenvironment	Tolerogenic: uNK cells, decidual macrophages, Tregs promote controlled invasion	Disrupted local immune regulation; decidual absence reduces immune ‘checkpoints’	Immunosuppressive tumor microenvironment, PD-L1 upregulation, and immune evasion pathways
ECM interactions	Balanced ECM degradation and deposition; fibronectin, laminin interactions	Excessive ECM breakdown with direct myometrial contact	ECM destruction, vasculogenic mimicry, intravascular tumor plugs
Angiogenesis	Physiological vascular remodeling, spiral artery transformation	Abnormal, poorly regulated angiogenesis leading to hemorrhage	Exuberant angiogenesis, vessel co-option, and neovascular networks
Clinical phenotype	Successful implantation, normal placentation	Morbidly adherent placenta → hemorrhage, surgical morbidity	Rapid systemic metastasis (lungs, brain, and liver), high mortality if untreated
Proliferation vs invasion balance	Low proliferation, high differentiation	Moderate proliferation, excessive invasion	High proliferation + malignant invasion
Molecular pathways			
MMP activity	Controlled	Elevated	Highly elevated
Integrins	α1β1, α5β1	Altered α/β profile	Dysregulated, promotes invasion/metastasis
hCG/H-hCG	Physiologic	Slightly elevated locally	Markedly elevated systemically
Angiogenesis	Controlled spiral artery remodeling	Abnormal, disorganized	Exuberant, including vasculogenic mimicry
Immune environment	Tolerogenic	Locally dysregulated	Immunosuppressive, PD-L1+, systemic escape
Proliferation	Low–moderate	Moderate	High, malignant

PAS, placenta accreta spectrum.

### Normal trophoblast invasion and uterine microenvironment

**Figure 1:**
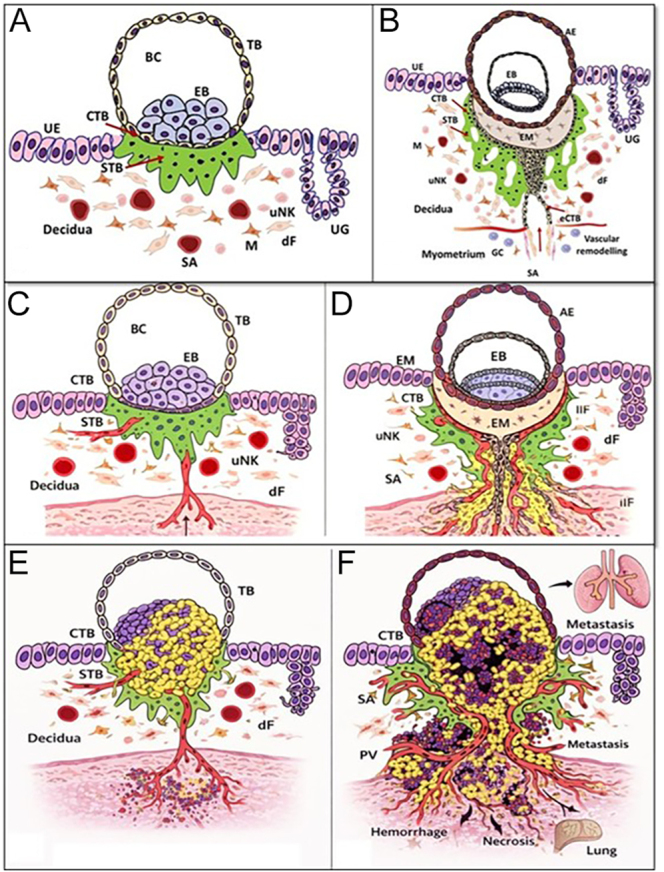
Schematic representation of trophoblast invasion in normal implantation, PAS, and choriocarcinoma. (A and B) Normal early human implantation. (A) Apposition and adhesion of the blastocyst to the uterine epithelium with differentiation of cytotrophoblast (CTB) and syncytiotrophoblast (STB), invasion limited to the decidua. (B) Controlled trophoblast invasion with formation of the extravillous trophoblast (EVT), physiological spiral artery (SA) remodeling, and preservation of the decidual barrier overlying the myometrium. (C and D) PAS. (C) Abnormally adherent placenta (placenta accreta) characterized by deficient decidua basalis, allowing direct attachment of anchoring villi and trophoblasts to the superficial myometrium. (D) Abnormally invasive placenta (placenta increta/percreta) showing excessive EVT invasion into the myometrium with exaggerated vascular remodeling and loss of normal uterine tissue planes. (E and F) Choriocarcinoma: (E) early invasive choriocarcinoma showing uncontrolled proliferation of CTB and STB without villous formation, with early vascular invasion, and (F) advanced choriocarcinoma demonstrating aggressive myometrial and vascular invasion, extensive hemorrhage and necrosis, and hematogenous metastasis, commonly to the lungs. (Panels A and B are original schematic illustrations manually created by one of the authors (RM) using conventional drawing methods. Panels C, D, E, and F were generated with the assistance of an AI-based image generation tool (OpenAI, GPT-5.3) using A and B as base references and subsequently reviewed and refined by the authors for scientific accuracy).

A synchronized interaction between the blastocyst and the maternal endometrium is necessary for successful human implantation, which leads to trophoblast invasion and placental development. Endometrium undergoes specific changes for implantation to occur, known as decidualization, which include modification of the endometrial stromal cells, uterine glands and vessels, as well as the increase in uterine immune cells (8). Decidualization regulates subsequent trophoblast invasion and placenta formation by changing the expression of important regulatory molecules, such as metalloproteinases, cytokines, surface integrins, and major histocompatibility complex molecules (9). Following blastocyst attachment, cytotrophoblasts differentiate into two major lineages: the multinucleated syncytiotrophoblast, responsible for nutrient exchange and endocrine functions, and the extravillous trophoblast (EVT), which invades the maternal decidua and remodels the spiral arteries to establish adequate uteroplacental perfusion (10). The EVT responds to signals from the decidua and releases paracrine factors that modulate gene expression in decidual cells, creating a dynamic bidirectional communication between the placenta and endometrium. Between weeks 8 and 20, trophoblast invasion occurs in a precisely regulated manner. EVTs migrate into the maternal decidua and myometrium and anchor villi to the uterine wall (11). These EVTs bring about the remodeling of spiral arteries to establish a low-resistance, high-capacitance uteroplacental circulation (12) (Fig. 1). This process corresponds to a tightly controlled epithelial–mesenchymal transition, characterized by downregulation of epithelial markers (E-cadherin) and overexpression of mesenchymal markers (N-cadherin and integrin α5β1). Unlike cancer invasion, this process is self-limiting and precisely regulated to balance fetal nutritional needs with maternal tissue preservation (1, 13).

The invasion process is guided by multiple molecular cues. Integrins, particularly the switch from α6β4 (epithelial) to α1β1 and α5β1 (mesenchymal), direct trophoblast adhesion and migration through the ECM (14). Matrix metalloproteinases (MMP-2 and MMP-9) mediate ECM degradation, while tissue inhibitors of metalloproteinases (TIMPs) restrain excessive proteolysis (15). Angiogenic factors, including VEGF, placental growth factor (PlGF), and hypoxia-inducible factors (HIFs), orchestrate vascular adaptation. Hyperglycosylated hCG (H-hCG) acts as an autocrine/paracrine factor promoting trophoblast invasiveness (16). The maternal decidua provides a permissive yet controlled environment for trophoblast invasion. The behavior of trophoblast is guided by the cytokines, chemokines, and angiogenic factors that are secreted by decidual stromal cells, uterine natural killer (uNK) cells, macrophages, and regulatory T-cells (17). Uterine natural killer (uNK) cells, which constitute up to 70% of leukocytes at the maternal–fetal interface, produce interferon-γ, angiogenic cytokines, and remodeling cytokines (VEGF and angiopoietin-2) rather than cytotoxic mediators promoting vascular remodeling while simultaneously restraining excessive trophoblast invasion (18). Decidual macrophages clear apoptotic cells and secrete growth factors, while T regulatory cells enforce immune tolerance to semi-allogeneic trophoblasts. A shift in Th1/Th2/Th17 balance ensures an environment permissive to invasion without provoking destructive inflammation. Importantly, EVT invasion is spatially restricted: it is deepest near the placental bed and tapers off toward the decidual periphery. Temporal control ensures that invasion peaks in the first trimester and stabilizes thereafter (19). This tightly regulated pattern prevents excessive trophoblastic penetration into the myometrium and maintains uterine structural integrity.

### Trophoblast invasion biology in PAS

The underlying biology of PAS reflects a dysregulation of the same trophoblastic mechanisms that mediate physiologic implantation and spiral artery remodeling (20). Normally, EVTs invade the decidua and superficial myometrium in a spatially and temporally controlled manner, guided by a balance between pro-invasive and anti-invasive molecular factors. In PAS, this equilibrium is disrupted, leading to exaggerated, deep, and uncontrolled invasion reminiscent of neoplastic behavior (21, 22). In normal placentation, the decidua basalis serves as a critical barrier modulating trophoblastic infiltration. The absence or attenuation of this decidual layer, often secondary to previous cesarean sections, curettage, or endometrial damage, removes an essential inhibitory interface (23). Histologically, PAS implantation sites lack the normal Nitabuch’s layer and exhibit direct attachment of anchoring villi to the myometrium (24, 25). This anatomic deficiency permits unchecked EVT penetration into the muscular wall and, in severe cases, through the serosa (26). Inflammation and immune tolerance pathways may also facilitate excessive trophoblast infiltration (27). EVTs in PAS display elevated PD-L1 and HLA-G expression, promoting local immune evasion and persistence within the myometrium (28).

At the molecular level, the invasive trophoblast phenotype in PAS mirrors a shift toward a more ‘pseudo-malignant’ behavior. Studies have shown upregulation of matrix metalloproteinases (MMP-2 and MMP-9), urokinase-type plasminogen activator (uPA), and integrins α1β1 and α5β1, all of which enhance extracellular matrix degradation and cellular motility (29). Conversely, regulatory molecules, such as tissue inhibitors of metalloproteinases (TIMPs), TGF-β, and decorin, are relatively downregulated, leading to loss of spatial control (30). Angiogenic signaling is also altered. Increased expression of vascular endothelial growth factor (VEGF) and placental growth factor (PlGF), along with reduced angiopoietin-1 levels, contributes to aberrant vascular remodeling and hypervascularity within the implantation site (30, 31, 32). Hypoxia-inducible factor-1α (HIF-1α) expression, normally transient during early implantation, persists abnormally in PAS, sustaining a pro-invasive trophoblastic phenotype (32). Moreover, the STAT3, p38, and JNK signaling cascades have been implicated in trophoblastic invasion in PAS, as FYN kinase promotes their activation through phosphorylation (33). Similarly, overexpression of LAMC2 in placental trophoblasts has been shown *in vitro* to enhance cell proliferation and migration, and LAMC2 contributes to PAS pathogenesis by triggering the PI3K/Akt/MMP2/9 signaling axis, thereby promoting excessive trophoblastic invasion (34). Another recently proposed mechanism contributing to the invasive trophoblast phenotype in PAS involves suppression of the Wnt–β-catenin/VEGF signaling pathway by pigment epithelium-derived factor (PEDF), which is notably downregulated in PAS tissues (35). Experimental data indicate that PEDF overexpression can inhibit EVT proliferation, invasion, and angiogenic activity, while inducing ferroptosis, a regulated form of cell death, thereby maintaining a balanced environment conducive to normal trophoblastic invasion.

**Figure 2:**
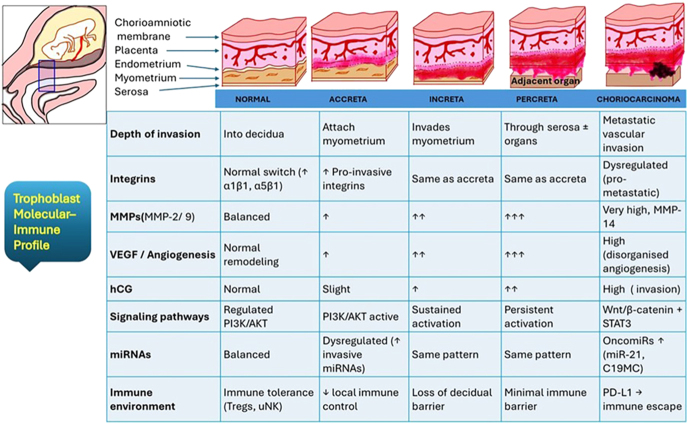
Comparative molecular and immune profile of trophoblast invasion across normal implantation, PAS, and choriocarcinoma. The schematic diagram illustrates progressive deepening of trophoblastic invasion and corresponding molecular changes in integrin expression, MMP activity, angiogenic signaling (VEGF and HIF-1α), and immune modulation (image created by one of the authors AS).

Epigenetic modulation may play a key role in PAS. Studies have identified aberrant methylation in promoter regions of invasion-related genes and dysregulated expression of microRNAs from the chromosome 19 microRNA cluster (C19MC), which normally act to restrain trophoblast invasion (36). In a study by Murrieta-Coxca *et al.* (37), multiple dysregulated miRNAs were identified in placenta accreta tissues. Specifically, miR-24-3p, miR-193b-3p, miR-331-3p, miR-376c-3p, miR-382-3p, miR-495-3p, miR-519d-3p, and miR-3074-5p were found to be upregulated, whereas miR-106b-3p, miR-222-3p, miR-370-3p, miR-454-5p, and miR-3615-3p were downregulated in PAS tissue. In addition, pathway enrichment analysis revealed a notable decrease in NF-κB mRNA levels, a finding corroborated in PAS samples, suggesting that miR-382-3p and miR-495-3p may contribute to enhanced trophoblast invasiveness through suppression of the NF-κB signaling pathway (37). Figure 2 represents the comparative molecular and immune profile of trophoblast invasion across normal implantation, PAS, and choriocarcinoma (Fig. 2).

### Invasive mole and choriocarcinoma: malignant invasion biology

An invasive mole, also known as chorioadenoma destruens, represents an intermediate entity in the spectrum of gestational trophoblastic disease (GTD), lying between the benign hydatidiform mole and the frankly malignant choriocarcinoma. It is characterized by persistent trophoblastic proliferation and local myometrial invasion by molar villi and trophoblastic cells yet retains villous structures unlike choriocarcinoma (38). Studies have demonstrated that key signaling pathways regulating trophoblast growth, migration, and differentiation are disrupted in invasive moles (39). In particular, aberrant activation of the Wnt/β-catenin pathway, which promotes epithelial-to-mesenchymal transition (EMT), imparts tumor-like invasive behavior to trophoblasts in complete hydatidiform moles. A major contributor to this invasiveness is the overexpression of matrix metalloproteinases (MMP-2 and MMP-9) along with reduced levels of tissue inhibitors of metalloproteinases (TIMPs). This MMP–TIMP imbalance, similar to that observed in choriocarcinoma, underlies the enhanced invasive potential of trophoblasts in invasive mole (39). Unlike PAS, however, invasion in an invasive mole is neoplastic rather than reactive, driven by intrinsic trophoblastic genetic alterations rather than secondary decidual deficiency.

In other words, the invasive mole exemplifies a ‘controlled malignancy’ within the GTD continuum, where the physiological mechanisms of trophoblast invasion become pathologically amplified yet remain histologically organized. In contrast, choriocarcinoma represents the most aggressive form of GTD, with early vascular invasion and widespread metastases (40). Unlike physiological trophoblasts, choriocarcinoma cells combine the natural invasive machinery of EVTs with malignant hallmarks, such as uncontrolled proliferation, genomic instability, and metastatic competence (41). Several molecular pathways are involved in the process of malignant invasiveness of choriocarcinoma (42). The malignant trophoblasts actively promote angiogenesis and enhance vascular permeability through the upregulation of angiogenic factors, such as VEGF-B (43, 44). The syncytiotrophoblast component secretes high levels of human chorionic gonadotropin (hCG), which not only serves as a clinical marker but also contributes to autocrine stimulation of trophoblast proliferation and immune modulation (45). Overexpression of MMPs, MMP-2, MMP-9, and MMP-14, facilitates ECM degradation and vascular infiltration (46). PI3K/AKT, Wnt/β-catenin, and Notch pathways are frequently activated, supporting proliferation, survival, and angiogenesis. Dysregulated miRNAs (e.g. miR-21 and miR-517a/c) enhance trophoblast invasiveness and immune evasion (47).

Recently, it has been found that choriocarcinoma creates an immunosuppressive tumor microenvironment, in part by upregulating PD-L1 on trophoblast-like cells and secreting immunomodulatory cytokines (48). This allows malignant trophoblasts to escape NK-cell-mediated cytotoxicity and T-cell surveillance, contrasting with the controlled immune permissiveness in normal implantation and the locally dysregulated immunity in PAS. Furthermore, a subpopulation of cancer stem-like cells within choriocarcinomas is believed to sustain invasion, recurrence, and therapeutic resistance, making anti-cancer stem cell approaches an emerging therapeutic avenue (49). Finally, epigenetic and noncoding RNA-mediated mechanisms, including long noncoding RNAs and microRNAs regulating the HIF3A axis, integrate hypoxic signaling with malignant transformation, thereby sustaining cell viability, proliferation, and invasion in this highly aggressive trophoblastic neoplasm. Noncoding RNAs, including members of the miR-302, miR-371a-5p, miR-518a-3p, and miR-519 families, normally active during early trophoblast and embryonic development show aberrant expression in choriocarcinoma (50). These dysregulated miRNAs have been implicated in cell cycle control, immune evasion, and metastatic progression (50).

Understanding the molecular pathways of trophoblastic invasion has implications for novel therapies, including targeted inhibitors of angiogenesis, immune checkpoint modulation, and MMP blockade. Recent advances in organoid cultures, xenografts, and single-cell transcriptomics have enabled a detailed study of choriocarcinoma invasion (51). These models reveal heterogeneity in invasive potential among trophoblastic cells, highlight pathways shared with normal trophoblasts, and identify candidate molecular targets for therapy (52). Such approaches bridge fundamental biology with translational applications, potentially guiding biomarker discovery and therapeutic innovation.

### Shared molecular pathways linking trophoblast invasion, vascular remodeling, and immune regulation

In normal placentation, invasion is tightly regulated with an intact decidua and preserved tissue planes. PAS shows loss of decidua basalis with exaggerated but benign trophoblastic invasion into the myometrium, increased angiogenesis and matrix remodeling, yet no cytological atypia or metastatic potential (25). In contrast, choriocarcinoma exhibit autonomous, destructive invasion with architectural distortion and cytological atypia. Despite distinct clinical phenotypes, several placental disorders share common molecular pathways that link trophoblast invasion with vascular remodeling, angiogenesis, and immune regulation at the maternal–fetal interface. PAS represents excessive trophoblast invasion, whereas conditions such as preeclampsia and fetal growth restriction are characterized by shallow invasion and impaired spiral artery remodeling; however, both extremes involve overlapping dysregulation of angiogenic signaling, extracellular matrix remodeling, and trophoblast–endothelial interactions (53, 54). Immune mechanisms, including altered crosstalk between extravillous trophoblasts and uterine immune cells, further contribute to disease pathogenesis. GTD, including choriocarcinoma, represents a malignant end of this spectrum, with exaggerated invasion and angiogenesis, highlighting a continuum of trophoblast-driven vascular and immune pathways across placental disorders.

### Models and methods to study trophoblast invasion

Models to study trophoblast invasion broadly include *in vitro*, *in vivo*, and *ex vivo* approaches. Cytotrophoblast and EVT cell lines have been extensively used to explore molecular pathways regulating invasion (55). *In vitro* models, such as EVT cell lines (HTR-8/SVneo and JEG-3) and placental organoids allow detailed study of migration, invasion, angiogenesis, and 3D cell–matrix interactions, enabling controlled manipulation of signaling pathways, such as HIF, TGF-β, Wnt, and Notch, providing high experimental control. Co-culture systems combining trophoblasts with decidual stromal cells, endothelial cells, or uterine natural killer (uNK) cells simulate the interactive cellular environment of the decidua. *Ex vivo* systems, including uterine explants of first-trimester placental villi or decidua, placental bed cultures, and decidua-mimetic hydrogels, preserve tissue architecture and enable investigation of trophoblast–decidua crosstalk and extracellular matrix remodeling but are often limited by viability and sample variability (56). Extravillous trophoblast (EVT) cell lines have been extensively utilized to study trophoblast differentiation, invasion, and interaction with the maternal decidua. While these properties make EVT cell lines conceptually relevant for PAS, their direct use as established *in vitro* models for PAS remains limited, with no standardized EVT-based PAS model currently available. In contrast, choriocarcinoma-derived trophoblastic cell lines (e.g. JEG-3, JAR, and BeWo) have been widely used and validated as experimental models for choriocarcinoma, particularly for studying tumor invasion, proliferation, and metastatic potential (25, 56, 57).

Animal models have provided crucial insights into trophoblast biology. *In vivo* models, such as mouse and rat implantation studies and xenograft choriocarcinoma models, offer systemic context and the ability to study PAS-like scarring, metastasis, vascular mimicry, and therapeutic interventions, although differences in human placentation can limit translational relevance (58). Advanced technologies further enhance the study of trophoblast invasion. Recent advances include trophoblast organoids derived from stem cells that self-organize into villous-like structures with hormone secretion and invasive capacities (59). Integration of single-cell and spatial transcriptomics technologies further enhances the understanding of trophoblast heterogeneity and invasion trajectories and provides detailed maps of cell populations and gene expression patterns across normal, PAS, and malignant tissues, while live imaging and microfluidics allow real-time visualization of invasion, angiogenesis, and cell–cell interactions (60). Each model has inherent strengths and limitations. *In vitro* systems offer precise molecular manipulation but lack systemic context; *ex vivo* and organoid approaches preserve architecture but have limited lifespan; and *in vivo* models provide physiological relevance but differ in placental structure and function from humans. Together, these complementary platforms form a robust toolkit for mechanistic studies and translational research in placental pathology and choriocarcinoma.

### Clinical and translational implications

The identification of reliable biomarkers holds major clinical potential for improving the diagnosis and management of both PAS and choriocarcinoma. PAS is diagnosed antenatally primarily by targeted obstetric ultrasonography with color Doppler, supported by MRI in equivocal or posterior placentation, while definitive diagnosis is confirmed histopathologically by the absence of decidua basalis with chorionic villi directly adherent to or invading the myometrium (25, 61). Serological diagnostic biomarkers for PAS remain largely adjunctive, with no single validated marker available for definitive prenatal diagnosis. Elevated maternal serum α-fetoprotein (AFP) is the most consistently reported association, thought to result from defective decidua basalis and increased fetomaternal leakage, although its specificity is limited (62). Increasing evidence implicates dysregulated angiogenesis in PAS, with altered expression of vascular endothelial growth factor (VEGF), placental growth factor (PlGF), and reduced soluble fms-like tyrosine kinase-1 (sFlt-1) favoring abnormal placental invasion (28). Abnormal levels of β-human chorionic gonadotropin (β-hCG) and pregnancy-associated plasma protein-A (PAPP-A) have also been described, reflecting excessive trophoblastic activity (62). In PAS, elevated H-hCG, MMPs, and integrins may serve as early predictors of abnormal placental invasion, enabling timely intervention to prevent hemorrhage and surgical morbidity (28). For choriocarcinoma, β-hCG and H-hCG remain diagnostic and monitoring standards, while novel circulating biomarkers, such as cfDNA, exosomal miRNAs, and trophoblast cells, offer prospects for earlier detection and treatment monitoring (63). A comparative summary of key biomarker alterations across these conditions is provided in Table 2.

**Table 2:** Comparative summary of biomarker alterations in normal placentation, PAS, and choriocarcinoma.

Biomarker/molecular pathway	Normal placentation	PAS	Choriocarcinoma
HIF-1α/oxygen sensing pathway	Transient physiological hypoxia maintains cytotrophoblast proliferation and angiogenesis in early gestation; declines with the establishment of maternal perfusion	Persistent HIF activation at scar sites promotes aberrant invasion and defective decidualization	Markedly upregulated; drives aggressive angiogenesis, proliferation, and hypoxia tolerance
VEGF/PlGF/sFlt-1 axis	Balanced VEGF–PlGF signaling ensures controlled vascular remodeling; normal sFlt-1 levels	↑ VEGF and ↓ E-cadherin locally enhance invasion; sFlt-1/PlGF ratio is usually normal systemically	Strongly elevated VEGF and PlGF expression; contributes to malignant vascular proliferation
MMP-2/MMP-9 (matrix metalloproteinases)	Controlled expression facilitates trophoblast invasion and spiral artery remodeling	Significantly upregulated MMP-9 and MMP-2 in invasive trophoblasts → excessive myometrial infiltration	Markedly overexpressed MMP-2/MMP-9 → extensive ECM degradation, metastasis
E-cadherin	Maintains epithelial integrity and regulates limited invasion	Downregulated at the implantation scar, reducing cell–cell adhesion and favoring excessive invasion	Severely downregulated; hallmark of epithelial–mesenchymal transition and metastasis
Syncytin-1/GCM1 pathway	Active trophoblast fusion and syncytiotrophoblast formation ensure endocrine and exchange function	Dysregulated syncytialization with persistent invasive cytotrophoblasts	Aberrant expression; loss of differentiation, predominance of proliferative cytotrophoblasts
β-hCG/hCG	Gradual physiologic rise; reflects normal syncytiotrophoblast activity	May be normal or mildly elevated due to increased local trophoblastic activity	Markedly elevated (>100,000 IU/L); diagnostic hallmark of trophoblastic malignancy
PAPP-A/PP13	Normal first-trimester levels support placental growth and vascular remodeling	May be reduced in defective decidualization or abnormal invasion	Not characteristically altered; nonspecific changes
Placental miRNA clusters (C19MC and C14MC)	Balanced expression regulates proliferation, invasion, and immune modulation	Dysregulated expression (↑ C19MC, ↓ C14MC) enhances trophoblast invasiveness	Markedly upregulated C19MC miRNAs promote oncogenic signaling and immune evasion
Immune tolerance markers (HLA-G, KIR/HLA-Cs)	Normal KIR/HLA-C interaction ensures controlled invasion and maternal tolerance	Loss of decidual immune control; defective decidualization and immune escape of invasive trophoblasts	Aberrant HLA-G expression confers immune evasion for malignant trophoblasts
Histopathologic correlate	Controlled invasion confined to the decidua basalis; intact Nitabuch’s layer	Villous tissue adherent or invading the myometrium with an absent decidual barrier	Diffuse malignant trophoblastic proliferation with hemorrhage, necrosis, and vascular invasion

PAS, placenta accreta spectrum.

Advances in imaging, including ultrasound, MRI, and emerging molecular imaging modalities, can further refine differentiation between normal, pathological, and malignant trophoblastic invasion and provide non-invasive assessment of invasion depth and vascular remodeling. From a translational standpoint, understanding the molecular drivers of invasion offers therapeutic promise. Agents targeting angiogenesis, MMPs, or immune checkpoints are being explored for choriocarcinoma, and similar insights could lead to preventive strategies for limiting excessive invasion in PAS (64). Comparative analyses across the invasion spectrum may yield shared targets and inform both obstetric and oncologic therapies. Liquid biopsy approaches, by enabling real-time tracking of invasive trophoblast activity, represent a key translational opportunity bridging diagnosis, prognosis, and therapeutic monitoring in both conditions.

### Knowledge gaps and future directions

Current research highlights critical knowledge gaps in understanding the continuum between physiological, pathological, and malignant trophoblastic invasion. It remains unclear which molecular pathways are shared between PAS and choriocarcinoma and which are unique to each process. Likewise, the mechanisms of immune modulation are incompletely understood. Local immune tolerance in PAS contrasts with the systemic immune evasion seen in choriocarcinoma. Additionally, the potential of circulating biomarkers, such as cfDNA or exosomal miRNAs, for early detection and differentiation of PAS and choriocarcinoma remains largely unexplored. Translational challenges also persist regarding the safe adaptation of anti-invasive or anti-angiogenic therapies within obstetric contexts. Future research should prioritize integrated multi-omics analyses combining genomic, transcriptomic, proteomic, and metabolomic data across normal placenta, PAS, and choriocarcinoma to delineate shared and divergent mechanisms. Development of advanced models, such as 3D trophoblastic organoids, immune co-culture systems, and humanized xenografts, will enhance mechanistic insight. Incorporating artificial intelligence to integrate imaging, molecular, and clinical parameters may facilitate predictive diagnostics. Ultimately, a cross-disciplinary framework bridging maternal–fetal medicine, oncology, and molecular biology will be essential to translate these findings into preventive, diagnostic, and therapeutic innovations.

## Conclusion

Trophoblast invasion represents a finely tuned balance between controlled cellular migration and immune modulation, essential for successful implantation and placental development. Disruption of this balance underlies a spectrum of pathologies, from insufficient invasion in preeclampsia to excessive infiltration in PAS disorders. Moreover, the molecular mechanisms that normally regulate trophoblast behavior can be hijacked in malignant contexts, such as choriocarcinoma, leading to aggressive and uncontrolled invasion. Understanding the cellular, molecular, and epigenetic pathways governing trophoblast invasion not only provides insight into normal reproductive physiology but also offers potential therapeutic targets for managing abnormal placentation and trophoblastic malignancies. Future research integrating genomics, proteomics, and advanced imaging promises to refine our understanding and improve outcomes for both maternal and fetal health.

## Declaration of interest

The authors declare that there are no conflicts of interest regarding the publication of this article.

## Funding

This work did not receive any specific grant from any funding agency in the public, commercial, or not-for-profit sector.

## Author contribution statement

MM, RM, and SP conceived the study and designed the outline; RM and AS created the schematic diagrams; MM, RM, SP, and AS and wrote the original draft of the manuscript; MM, RM, SP, and AS reviewed the manuscript; MM, RM, and SP supervised the overall preparation and critical revision of the manuscript.

## AI declaration

Figure 1 – panels A and B are original schematic illustrations manually created by one of the authors (RM) using conventional drawing methods. Panels C, D, E, and F were generated with the assistance of an AI-based image generation tool (OpenAI, GPT-5.3) using panels A and B as base references and subsequently reviewed and refined by the authors for scientific accuracy. All images are original schematic representations created specifically for this manuscript, do not reproduce or derive from any copyrighted material, and do not include any patient-specific data.

## References

[1] Moser G, Windsperger K, Pollheimer J, et al. Human trophoblast invasion: new and unexpected routes and functions. Histochem Cell Biol 2018 150 361–370. (10.1007/s00418-018-1699-0) 30046889 PMC6153604

[2] Park JY, Mani S, Clair G, et al. A microphysiological model of human trophoblast invasion during implantation. Nat Commun 2022 13 1252. (10.1038/s41467-022-28663-4) 35292627 PMC8924260

[3] Afshar Y, Kashani Ligumsky L, Bartels HC, et al. Biology and pathophysiology of Placenta accreta spectrum disorder. Obstet Gynecol 2025 145 611–620. (10.1097/AOG.0000000000005903) 40209229 PMC12068549

[4] Seckl MJ, Sebire NJ & Berkowitz RS. Gestational trophoblastic disease. Lancet 2010 376 717–729. (10.1016/S0140-6736(10)60280-2) 20673583

[5] Ferretti C, Bruni L, Dangles-Marie V, et al. Molecular circuits shared by placental and cancer cells, and their implications in the proliferative, invasive, and migratory capacities of trophoblasts. Hum Reprod Update 2007 13 121–141. (10.1093/humupd/dml048) 17068222

[6] Kohi MP, Rizzuto GA, Fidelman N, et al. Retained placenta accreta mimicking choriocarcinoma. Case Rep Pathol 2015 2015 167986. (10.1155/2015/167986) 26495146 PMC4606209

[7] Morlando M & Collins S. Placenta accreta spectrum disorders: challenges, risks, and management strategies. Int J Womens Health 2020 12 1033–1045. (10.2147/IJWH.S224191) 33204176 PMC7667500

[8] Salamonsen LA, Dimitriadis E, Jones RL, et al. Complex regulation of decidualization: a role for cytokines and proteases – a review. Placenta 2003 24 (Supplement A) 76–85. (10.1053/plac.2002.0928) 12842418

[9] Hess AP, Hamilton AE, Talbi S, et al. Decidual stromal cell response to para crine signals from the trophoblast: amplification of immune and angiogenic modulators. Biol Reprod 2007 76 102–117. (10.1095/biolreprod.106.054791) 17021345

[10] Huppertz B. The anatomy of the normal placenta. J Clin Pathol 2008 61 1296–1302. (10.1136/jcp.2008.055277) 18755720

[11] Pijnenborg R, Vercruysse L & Hanssens M. The uterine spiral arteries in human pregnancy: facts and controversies. Placenta 2006 27 939–958. (10.1016/j.placenta.2005.12.006) 16490251

[12] Lunghi L, Ferretti ME, Medici S, et al. Control of human trophoblast function. Reprod Biol Endocrinol 2007 5 6. (10.1186/1477-7827-5-6) 17288592 PMC1800852

[13] Cheng JC, Chang HM & Leung PC. Transforming growth factor-β1 inhibits trophoblast cell invasion by inducing Snail-mediated down-regulation of vascular endothelial-cadherin protein. J Biol Chem 2013 288 33181–33192. (10.1074/jbc.M113.488866) 24106276 PMC3829165

[14] Anin SA, Vince G & Quenby S. Trophoblast invasion. Hum Fertil 2004 7 169–174. (10.1080/14647270400006911) 15590570

[15] Staun-Ram E & Shalev E. Human trophoblast function during the implantation process. Reprod Biol Endocrinol 2005 3 56. (10.1186/1477-7827-3-56) 16236179 PMC1289292

[16] Huppertz B. Traditional and new routes of trophoblast invasion and their implications for pregnancy diseases. Int J Mol Sci 2019 21 289. (10.3390/ijms21010289) 31906245 PMC6981830

[17] Nancy P & Erlebacher A. T cell behavior at the maternal-fetal interface. Int J Dev Biol 2014 58 189–198. (10.1387/ijdb.140054ae) 25023685 PMC4212519

[18] Parham P. NK cells and trophoblasts: partners in pregnancy. J Exp Med 2004 200 951–955. (10.1084/jem.20041783) 15492121 PMC2211836

[19] Gerbaud P, Taskén K & Pidoux G. Spatiotemporal regulation of cAMP signaling controls the human trophoblast fusion. Front Pharmacol 2015 6 202. (10.3389/fphar.2015.00202) 26441659 PMC4569887

[20] Illsley NP, DaSilva-Arnold SC, Zamudio S, et al. Trophoblast invasion: lessons from abnormally invasive placenta (placenta accreta). Placenta 2020 102 61–66. (10.1016/j.placenta.2020.01.004) 33218581 PMC7680503

[21] Jauniaux E, Aplin JD, Fox KA, et al. Placenta accreta spectrum. Nat Rev Dis Primers 2025 11 40. (10.1038/s41572-025-00624-3) 40473652

[22] Jansen C, Kastelein AW, Kleinrouweler CE, et al. Development of placental abnormalities in location and anatomy. Acta Obstet Gynecol Scand 2020 99 983–993. (10.1111/aogs.13834) 32108320 PMC7496588

[23] Jauniaux E & Jurkovic D. Placenta accreta: pathogenesis of a 20th century iatrogenic uterine disease. Placenta 2012 33 244–251. (10.1016/j.placenta.2011.11.010) 22284667

[24] Mangla M, Palo S & Motwani R. Nitabuch’s and Rohr’s fibrinoid layers: revisiting the interface of the placenta and uterus. J Obstet Gynaecol Res 2025 51 e70052. (10.1111/jog.70052) 40841330

[25] Hecht JL, Baergen R, Ernst LM, et al. Classification and reporting guidelines for the pathology diagnosis of placenta accreta spectrum (PAS) disorders: recommendations from an expert panel. Mod Pathol 2020 33 2382–2396. (10.1038/s41379-020-0569-1) 32415266

[26] Jauniaux E & Burton GJ. Pathophysiology of Placenta accreta spectrum disorders: a review of current findings. Clin Obstet Gynecol 2018 61 743–754. (10.1097/grf.0000000000000392) 30299280

[27] Cheng SB, Nakashima A, Huber WJ, et al. Pyroptosis is a critical inflammatory pathway in the placenta from early onset preeclampsia and in human trophoblasts exposed to hypoxia and endoplasmic reticulum stressors. Cell Death Dis 2019 10 927. (10.1038/s41419-019-2162-4) 31804457 PMC6895177

[28] Bartels HC, Postle JD, Downey P, et al. Placenta accreta spectrum: a review of pathology, molecular biology, and biomarkers. Dis Markers 2018 2018 1507674. (10.1155/2018/1507674) 30057649 PMC6051104

[29] Gualdoni G, Gomez Castro G, Hernández R, et al. Comparative matrix metalloproteinase-2 and -9 expression and activity during endotheliochorial and hemochorial trophoblastic invasiveness. Tissue Cell 2022 74 101698. (10.1016/j.tice.2021.101698) 34871824

[30] Lizárraga-Verdugo E, Beltrán-Ontiveros SA, Gutiérrez-Grijalva EP, et al. The underlying molecular mechanisms of the placenta accreta spectrum: a narrative review. Int J Mol Sci 2024 25 9722. (10.3390/ijms25179722) 39273667 PMC11395310

[31] Arakaza A, Liu X, Zhu J, et al. Assessment of serum levels and placental bed tissue expression of IGF-1, bFGF, and PLGF in patients with placenta previa complicated with placenta accreta spectrum disorders. J Matern Fetal Neonatal Med 2024 37 2305264. (10.1080/14767058.2024.2305264) 38247274

[32] Schwickert A, Chantraine F, Ehrlich L, et al. Maternal serum VEGF predicts abnormally invasive placenta better than NT-proBNP: a multicenter case-control study. Reprod Sci 2021 28 361–370. (10.1007/s43032-020-00319-y) 33025531 PMC7808970

[33] Liu M, Su C, Zhu L, et al. Highly expressed FYN promotes the progression of placenta accreta by activating STAT3, p38, and JNK signaling pathways. Acta Histochem 2023 125 151991. (10.1016/j.acthis.2022.151991) 36563468

[34] Wang R, Liu W, Zhao J, et al. Overexpressed LAMC2 promotes trophoblast over-invasion through the PI3K/Akt/MMP2/9 pathway in placenta accreta spectrum. J Obstet Gynaecol Res 2023 49 548–559. (10.1111/jog.15493) 36412218

[35] Li R, Weng X, Hu X, et al. Pigment epithelium-derived factor inhibits proliferation, invasion and angiogenesis, and induces ferroptosis of extravillous trophoblasts by targeting Wnt-β-catenin/VEGF signaling in placenta accreta spectrum. Mol Med Rep 2024 29 75. (10.3892/mmr.2024.13199) 38488028 PMC10975022

[36] Kohan-Ghadr HR, Kadam L, Jain C, et al. Potential role of epigenetic mechanisms in regulation of trophoblast differentiation, migration, and invasion in the human placenta. Cell Adh Migr 2016 10 126–135. (10.1080/19336918.2015.1098800) 26745760 PMC4853046

[37] Murrieta-Coxca JM, Barth E, Fuentes-Zacarias P, et al. Identification of altered miRNAs and their targets in placenta accreta. Front Endocrinol 2023 14 1021640. (10.3389/fendo.2023.1021640) PMC10022468 36936174

[38] Akyol A, Şimşek M & Üçer Ö. Giant invasive mole presenting as a cause of abdominopelvic mass in a perimenopausal woman: an unusual presentation of a rare pathology. Obstet Gynecol Sci 2016 59 548–553. (10.5468/ogs.2016.59.6.548) 27896261 PMC5120078

[39] Mustafa S, Koran S & AlOmair L. Insights into the role of matrix metalloproteinases in cancer and its various therapeutic aspects: a review. Front Mol Biosci 2022 9 896099. (10.3389/fmolb.2022.896099) 36250005 PMC9557123

[40] Cheung AN, Zhang HJ, Xue WC, et al. Pathogenesis of choriocarcinoma: clinical, genetic and stem cell perspectives. Future Oncol 2009 5 217–231. (10.2217/14796694.5.2.217) 19284380

[41] Camilleri G, Calleja-Aguis J & Said E. Trophoblastic disease and choriocarcinoma. Eur J Surg Oncol 2025 51 108727. (10.1016/j.ejso.2024.108727) 39370364

[42] Nicheperovich A, Schuster-Böckler B & Ní Leathlobhair M. Gestational trophoblastic disease: understanding the molecular mechanisms of placental tumours. Dis Model Mech 2025 18 DMM052010. (10.1242/dmm.052010) 39873178 PMC11810044

[43] Shih IM. Trophoblastic vasculogenic mimicry in gestational choriocarcinoma. Mod Pathol 2011 24 646–652. (10.1038/modpathol.2010.231) 21217646

[44] Xue Y, Sun R, Zheng W, et al. Forskolin promotes vasculogenic mimicry and invasion via Notch-1-activated epithelial-to-mesenchymal transition in syncytiolization of trophoblast cells in choriocarcinoma. Int J Oncol 2020 56 1129–1139. (10.3892/ijo.2020.4997) 32319581 PMC7115352

[45] Cole LA, Butler SA, Khanlian SA, et al. Gestational trophoblastic diseases: 2. Hyperglycosylated hCG as a reliable marker of active neoplasia. Gynecol Oncol 2006 102 151–159. (10.1016/j.ygyno.2005.12.045) 16631241

[46] Nurseta T, Indrawan IWA, Gunawan DA, et al. Effects atra on MMP-9 activity and integrin expression in choriocarcinoma culture cell line bewo (ATCC CCL-98). Asian Pac J Cancer Prev 2025 26 1027–1033. (10.31557/APJCP.2025.26.3.1027) 40156421 PMC12174550

[47] Jung SH, Choi YJ, Kim MS, et al. Distinct genomic profiles of gestational choriocarcinoma, a unique cancer of pregnant tissues. Exp Mol Med 2020 52 2046–2054. (10.1038/s12276-020-00544-0) 33319857 PMC8080714

[48] Reynaud D, Abi Nahed R, Lemaitre N, et al. NLRP7 promotes choriocarcinoma growth and progression through the establishment of an immunosuppressive microenvironment. Cancers 2021 13 2999. (10.3390/cancers13122999) 34203890 PMC8232770

[49] Cai J, Peng T, Wang J, et al. Isolation, culture and identification of choriocarcinoma stem-like cells from the human choriocarcinoma cell-line JEG-3. Cell Physiol Biochem 2016 39 1421–1432. (10.1159/000447845) 27606814

[50] Murray MJ, Smith S, Ward D, et al. Circulating microRNAs as biomarkers to assist the management of the malignant germ-cell-tumour subtype choriocarcinoma. Transl Oncol 2021 14 100904. (10.1016/j.tranon.2020.100904) 33049521 PMC7557903

[51] Grümmer R, Hohn HP, Mareel MM, et al. Adhesion and invasion of three human choriocarcinoma cell lines into human endometrium in a three-dimensional organ culture system. Placenta 1994 15 411–429. (10.1016/0143-4004(94)90008-6) 7937597

[52] Liu Y, Wu W, Cai C, et al. Patient-derived xenograft models in cancer therapy: technologies and applications. Signal Transduct Target Ther 2023 8 160. (10.1038/s41392-023-01419-2) 37045827 PMC10097874

[53] Liu Y, Fan X, Wang R, et al. Single-cell RNA-seq reveals the diversity of trophoblast subtypes and patterns of differentiation in the human placenta. Cell Res 2018 28 819–832. (10.1038/s41422-018-0066-y) 30042384 PMC6082907

[54] Lv B, An Q, Zeng Q, et al. Single-cell RNA sequencing reveals regulatory mechanism for trophoblast cell-fate divergence in human peri-implantation conceptuses. PLoS Biol 2019 17 e3000187. (10.1371/journal.pbio.3000187) 31596842 PMC6802852

[55] Graham CH, Hawley TS, Hawley RC, et al. Establishment and characterization of first trimester human trophoblast cells with extended lifespan. Exp Cell Res 1993 206 204–211. (10.1006/excr.1993.1139) 7684692

[56] Carter AM. Animal models of human placentation – a review. Placenta 2007 28 (Supplement A) S41–S47. (10.1016/j.placenta.2006.11.002) 17196252

[57] King A, Thomas L & Bischof P. Cell culture models of trophoblast II: trophoblast cell lines – a workshop report. Placenta 2000 21 (Supplement A) S113–S119. (10.1053/plac.1999.0526) 10831135

[58] Soncin F, Natale D & Parast MM. Signaling pathways in mouse and human trophoblast differentiation: a comparative review. Cell Mol Life Sci 2015 72 1291–1302. (10.1007/s00018-014-1794-x) 25430479 PMC4366325

[59] Sheridan MA, Fernando RC, Gardner L, et al. Establishment and differentiation of long-term trophoblast organoid cultures from the human placenta. Nat Protoc 2020 15 3441–3463. (10.1038/s41596-020-0381-x) 32908314

[60] Vento-Tormo R, Efremova M, Botting RA, et al. Single-cell reconstruction of the early maternal-fetal interface in humans. Nature 2018 563 347–353. (10.1038/s41586-018-0698-6) 30429548 PMC7612850

[61] Patel-Lippmann KK, Planz VB, Phillips CH, et al. Placenta accreta spectrum disorders: update and pictorial review of the SAR-ESUR joint consensus statement for MRI. Radiographics 2023 43 e220090. (10.1148/rg.220090) 37079459

[62] Givens M, Valcheva I, Einerson BD, et al. Evaluation of maternal serum protein biomarkers in the prenatal evaluation of placenta accreta spectrum: a systematic scoping review. Acta Obstet Gynecol Scand 2024 103 2335–2347. (10.1111/aogs.14918) 39004916 PMC11610010

[63] Peng X, Zhang Z, Mo Y, et al. Bioinformatics analysis of choriocarcinoma-related MicroRNA-transcription factor-target gene regulatory networks and validation of key miRNAs. Onco Targets Ther 2021 14 3903–3919. (10.2147/ott.s311291) 34234459 PMC8254590

[64] Song Y, Fu Y, Xie Q, et al. Anti-angiogenic agents in combination with immune checkpoint inhibitors: a promising strategy for cancer treatment. Front Immunol 2020 11 1956. (10.3389/fimmu.2020.01956) 32983126 PMC7477085

